# The Connection of Social Services to Care Transitions and Discharge Planning in Nursing Home Settings

**DOI:** 10.1093/geroni/igaa057.2536

**Published:** 2020-12-16

**Authors:** Colleen Galambos, Laura Rollin, Eric Engelbart

**Affiliations:** University of Wisconsin Milwaukee Helen Bader School of Social Welfare, Milwaukee, Wisconsin, United States

## Abstract

Care transitions are critical junctures in the healthcare delivery process. Effective transitions and discharge planning reduce the need for subsequent transfers between healthcare settings (Boutwell et al., 2015). Understanding social services (SS) involvement in these processes is important due to its key role in their success (Fabbre et al., 2011). Facility characteristics from 924 nursing homes were evaluated in relation to SS involvement in care transitions and discharge planning. Chi-square tests indicate associations between SS involvement and level of engagement of SS expertise by the nursing home administrator (p=.004), medical director (p=.002), nursing staff (p=.003), community physicians (p=.049), and family members (p<.001). An association between SS involvement and freestanding SS departments was also observed. Results suggest the level of SS involvement in care transitions and discharge planning relates to structural (i.e. SS positioning within the facility) and relational (i.e. perceptions and utilization of SS designees by key facility leadership) factors.

